# KnowLife: a versatile approach for constructing a large knowledge graph for biomedical sciences

**DOI:** 10.1186/s12859-015-0549-5

**Published:** 2015-05-14

**Authors:** Patrick Ernst, Amy Siu, Gerhard Weikum

**Affiliations:** Max-Planck-Institute for Informatics, Campus E1 4, Saarbrücken, 66123 Germany

**Keywords:** Biomedical text mining, Knowledge base, Relation extraction

## Abstract

**Background:**

Biomedical knowledge bases (KB’s) have become important assets in life sciences. Prior work on KB construction has three major limitations. First, most biomedical KBs are manually built and curated, and cannot keep up with the rate at which new findings are published. Second, for automatic information extraction (IE), the text genre of choice has been scientific publications, neglecting sources like health portals and online communities. Third, most prior work on IE has focused on the molecular level or chemogenomics only, like protein-protein interactions or gene-drug relationships, or solely address highly specific topics such as drug effects.

**Results:**

We address these three limitations by a versatile and scalable approach to automatic KB construction. Using a small number of seed facts for distant supervision of pattern-based extraction, we harvest a huge number of facts in an automated manner without requiring any explicit training.

We extend previous techniques for pattern-based IE with confidence statistics, and we combine this recall-oriented stage with logical reasoning for consistency constraint checking to achieve high precision. To our knowledge, this is the first method that uses consistency checking for biomedical relations. Our approach can be easily extended to incorporate additional relations and constraints.

We ran extensive experiments not only for scientific publications, but also for encyclopedic health portals and online communities, creating different KB’s based on different configurations. We assess the size and quality of each KB, in terms of number of facts and precision. The best configured KB, KnowLife, contains more than 500,000 facts at a precision of 93% for 13 relations covering genes, organs, diseases, symptoms, treatments, as well as environmental and lifestyle risk factors.

**Conclusion:**

KnowLife is a large knowledge base for health and life sciences, automatically constructed from different Web sources. As a unique feature, KnowLife is harvested from different text genres such as scientific publications, health portals, and online communities. Thus, it has the potential to serve as one-stop portal for a wide range of relations and use cases. To showcase the breadth and usefulness, we make the KnowLife KB accessible through the health portal (http://knowlife.mpi-inf.mpg.de).

**Electronic supplementary material:**

The online version of this article (doi:10.1186/s12859-015-0549-5) contains supplementary material, which is available to authorized users.

## Introduction

Large knowledge bases (KB’s) about entities, their properties, and the relationships between entities, have become an important asset for semantic search, analytics, and smart recommendations over Web contents and other kinds of Big Data [1,2]. Notable projects are DBpedia [3], Yago [4], and the Google Knowledge Graph with its public core Freebase (freebase.com).

In the biomedical domain, KB’s such as the Gene Ontology, the Disease Ontology, the National Drug File - Reference Terminology, and the Foundational Model of Anatomy are prominent examples of the rich knowledge that is digitally available. However, each of these KB’s is highly specialized and covers only a relative narrow topic within the life sciences, and there is very little interlinkage between the KB’s. Thus, in contrast to the general-domain KB’s that power Web search and analytics, there is no way of obtaining an integrated view on all aspects of biomedical knowledge. The lack of a “one-stop” KB that spans biological, medical, and health knowledge, hinders the development of advanced search and analytic applications in this field.

In order to build a comprehensive biomedical KB, the following three bottlenecks must be addressed.

**Beyond manual curation.** Biomedical knowledge is advancing at rates far greater than any single human can absorb. Therefore, relying on manual curation of KB’s is bound to be a bottleneck. To fully leverage all published knowledge, automated information extraction (IE) from input texts is mandatory.

**Beyond scientific literature.** Besides scientific publications found in PubMed Medline and PubMed Central, there are substantial efforts on patient-oriented health portals such as Mayo Clinic, Medline Plus, UpToDate, Wikipedia’s Health Portal, and there are also popular online discussion forums such as healthboards.com or patient.co.uk. All this constitutes a rich universe of information, but the information is spread across many sources, mostly in textual, unstructured and sometimes noisy form. Prior work on biomedical IE has focused on scientific literature only, and completely disregards the opportunities that lie in tapping into health portals and communities for automated IE.

**Beyond molecular entities.** IE from biomedical texts has strongly focused on entities and relations at the molecular level; a typical IE task is to extract protein-protein interactions. There is very little work on comprehensive approaches that link diverse entity types, spanning genes, diseases, symptoms, anatomic parts, drugs, drug effects, etc. In particular, no prior work on KB construction has addressed the aspects of environmental and lifestyle risk factors in the development of diseases and the effects of drugs and therapies.

## Background

The main body of IE research in biomedical informatics has focused on molecular entities and chemogenomics, like Protein-Protein Interactions (PPI) or gene-drug relations. These efforts have been driven by competitions such as BioNLP Shared Task (BioNLP-ST) [5] and BioCreative [6]. These shared tasks come with pre-annotated corpora as gold standard, such as the GENIA corpus [7], the multi-level event extraction (MLEE) corpus [5], and various BioCreative corpora. Efforts such as the Pharmacogenetics Research Network and Knowledge Base (PharmGKB) [8], which curates and disseminates knowledge about the impact of human genetic variations on drug responses, or the Open PHACTS project [9], a pharmacological information platform for drug discovery, offer knowledge bases with annotated text corpora to facilitate approaches for these use cases.

Most IE work in this line of research relies on supervised learning, like Support Vector Machines [10-13] or Probabilistic Graphical Models [14,15]. The 2012 i2b2 challenge aimed at extracting temporal relations from clinical narratives [16]. Unsupervised approaches have been pursued by [17-20], to discover associations between genes and diseases based on the co-occurrence of entities as cues for relations. To further improve the quality of discovered associations, crowdsourcing has also been applied [21,22]. Burger et al. [23] uses Amazon Mechanical Turk to validate gene-mutation relations which are extracted from PubMed abstracts. Aroyo et al. [24] describes a crowdsourcing approach to generate gold standard annotations for medical relations, taking into account the disagreement between crowd workers.

Pattern-based approaches exploit text patterns that connect entities. Many of them [25-28] manually define extraction patterns. Kolářik et al. [29] uses Hearst patterns [30] to identify terms that describe various properties of drugs. SemRep [31] manually specifies extraction rules obtained from dependency parse trees. Outside the biomedical domain, sentic patterns [32] leverage commonsense and syntactic dependencies to extract sentiments from movie reviews. However, while manually defined patterns yield high precision, they rely on expert guidance and do not scale to large and potentially noisy inputs and a broader scope of relations. Bootstrapping approaches such as [33,34] use a limited number of seeds to learn extraction patterns; these techniques go back to [35,36]. Our method follows this paradigm, but extends prior work with additional statistics to quantify the confidence of patterns and extracted facts.

A small number of projects like Sofie/Prospera [37,38] and NELL [39] have combined pattern-based extraction with logical consistency rules that constrain the space of fact candidates. Nebot et al. [40] harness the IE methods of [38] for populating disease-centric relations. This approach uses logical consistency reasoning for high precision, but the small scale of this work leads to a very restricted KB. Movshovitz-Attias et al. [41] used NELL to learn instances of biological classes, but did not extract binary relations and did not make use of constraints either. The other works on constrained extraction tackle non-biological relations only (e.g., birthplaces of people or headquarters of companies). Our method builds on Sofie/Prospera, but additionally develops customized constraints for the biomedical relations targeted here.

Most prior work in biomedical Named Entity Recognition (NER) specializes in recognizing specific types of entities such as proteins and genes, chemicals, diseases, and organisms. MetaMap [42] is the most notable tool capable of recognizing a wide range of entities. As for biomedical Named Entity Disambiguation (NED), there is relatively little prior work. MetaMap offers limited NED functionality, while others focus on disambiguating between genes [43] or small sets of word senses [44].

Most prior IE work processes only abstracts of Pubmed articles; few projects have considered full-length articles from Pubmed Central, let alone Web portals and online communities. Vydiswaran et al. [45] addressed the issue of assessing the credibility of medical claims about diseases and their treatments in health portals. Mukherjee et al. [46] tapped discussion forums to assess statements about side effects of drugs. White et al. [47] demonstrated how to derive insight on drug effects from query logs of search engines. Building a comprehensive KB from such raw assets has been beyond the scope of these prior works.

## Contributions

We present KnowLife, a large KB that captures a wide variety of biomedical knowledge, automatically extracted from different genres of input sources. KnowLife’s novel approach to KB construction overcomes the following three limitations of prior work.

**Beyond manual curation.** Using distant supervision in the form of seed facts from existing expert-level knowledge collections, the KnowLife processing pipeline is able to automatically learn textual patterns and harvest a large number of relational facts from such patterns. In contrast to prior work on IE for biomedical data which relies on extraction patterns only, our method achieves high precision by specifying and checking logical consistency constraints that fact candidates have to satisfy. These constraints are customized for the relations of interest in KnowLife, and include constraints that couple different relations. The consistency constraints are available as supplementary material (see Additional file 1). KnowLife is easily extensible, since new relations can be added with little manual effort and without requiring explicit training; only a small number of seed facts for each new relation is needed.

**Beyond scientific literature.** KnowLife copes with input text at large scale – considering not only knowledge from scientific publications, but also tapping into previously neglected textual sources like Web portals on health issues and online communities with discussion boards. We present an extensive evaluation of 22,000 facts on how these different genres of input texts affect the resulting precision and recall of the KB. We also present an error analysis that provides further insight on the quality and contribution of different text genres.

**Beyond molecular entities.** The entities and facts in KnowLife go way beyond the traditionally covered level of proteins and genes. Besides genetic factors of diseases, the KB also captures diseases, therapies, drugs, and risk factors like nutritional habits, life-style properties, and side effects of treatments.

In summary, the novelty of KnowLife is its versatile, largely automated, and scalable approach for the comprehensive construction of a KB – covering a spectrum of different text genres as input and distilling a wide variety facts from different biomedical areas as output. Coupled with an entity recognition module that covers the entire range of biomedical entities, the resulting KB features a much wider spectrum of knowledge and use-cases than previously built, highly specialized KB’s. In terms of methodology, our extraction pipeline builds on existing techniques but extends them, and is specifically customized to the life-science domain. Most notably, unlike prior work on biomedical IE, KnowLife employs logical reasoning for checking consistency constraints, tailored to the different relations that connect diseases, symptoms, drugs, genes, risk factors, etc. This constraint checking eliminates many false positives that are produced by methods that solely rely on pattern-based extraction.

In its best configuration, the KnowLife KB contains a total of 542,689 facts for 13 different relations, with an average precision of 93% (i.e., validity of the acquired facts) as determined by extensive sampling with manual assessment. The precision for the different relations ranges from 71% (createsRisk: ecofactor × disease) to 97% (sideEffect:(symptom ∪ disease) × drug). All facts in KnowLife carry provenance information, so that one can explore the evidence for a fact and filter by source. We developed a web portal that showcases use-cases from speed-reading to semantic search along with richly annotated literature, the details of which are described in the demo paper [48].

## Methods

**Figure 1:**
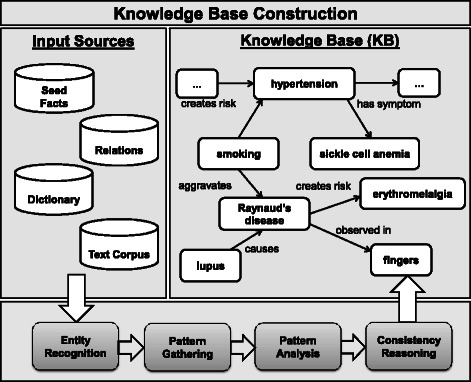
Overview of the KnowLife KB and processing pipeline.

Our method for harvesting relational facts from text sources is designed as a pipeline of processing stages; Figure 1 gives a pictorial overview. A fact is a triple consisting of two entities *e*_1_,*e*_2_ and a relation *R* between them; we denote a fact by *R*(*e*_1_,*e*_2_). In the following, we describe the input data and each stage of the pipeline.

### Input sources

**Dictionary** We use UMLS (Unified Medical Language System) as the dictionary of biomedical entities. UMLS is a metathesaurus, the largest collection of biomedical dictionaries containing 2.9 million entities and 11.4 million entity names and synonyms. Each entity has a *semantic type* assigned by experts. For instance, the entities *IL4R* and *asthma* are of semantic types *Gene or Genome* and *Disease or Syndrome*, respectively. The UMLS dictionary enables KnowLife to detect entities in text, going beyond genes and proteins and covering entities about anatomy, physiology, and therapy.

**Relations** KnowLife currently supports 13 binary relations between entities, each with a type signature constraining its domain and range (i.e., its left and right argument types). Table 1 shows that, for instance, the relation *affects* only holds between diseases and organs, but not between diseases and drugs. Each type signature consists of multiple fine-grained semantic types defined by UMLS; specifics for all relations are provided as supplementary material (see Additional file 2).

**Table 1:** **KnowLife relations, their type signatures, and number of seeds**

**Relation**	**Domain**	**Range**	**Seed facts**
Affects	Disease	Organ	23
Aggravates	Ecofactor	Disease	21
Alleviates	Drug	Disease	18
Causes	Disease	Disease	70
ComplicationOf	Disease	Disease	5
Contraindicates	Drug	Disease	26
CreatesRisk	Ecofactor	Disease	103
Diagnoses	Device	Disease	29
Interacts	Drug	Drug	9
IsSymptom	Symptom or Disease	Disease	69
ReducesRisk	Drug or Behavior	Disease	24
SideEffect	Symptom or Disease	Drug	12
Treats	Drug	Disease	58

**Seed facts.** A *seed fact* *R*(*e*_1_,*e*_2_) for relation *R* is a triple presumed to be true based on expert statements. We collected 467 seed facts (see Table 1) from the medical online portal uptodate.com, a highly regarded clinical resource written by physician authors. These seed facts are further cross-checked in other sources to assert their veracity. Example seed facts include *i* *s* *S* *y* *m* *p* *t* *o* *m*(*C* *h* *e* *s* *t* *P* *a* *i* *n*,*M* *y* *o* *c* *a* *r* *d* *i* *a* *l* *I* *n* *f* *a* *r* *c* *t* *i* *o* *n*) and *c* *r* *e* *a* *t* *e* *s* *R* *i* *s* *k*(*O* *b* *e* *s* *i* *t* *y*,*D* *i* *a* *b* *e* *t* *e* *s*).

**Text Corpus.** A key asset of this work is that we tap into different genres of text; Table 2 gives an overview. PubMed documents are scientific texts with specialized jargon; they have been the de-facto standard corpus for biomedical text mining. We took all PubMed documents published in 2011 that are indexed with disease-, drug-, and therapy-related MeSH (Medical Subject Heading) terms. We further prune out documents from inapplicable journals such as those not in the English language, or those about medical ethics. Web portals and encyclopedic articles are collaboratively or professionally edited, providing credible information in layman-oriented language. Examples include uptodate.com, mayoclinic.com, and the relevant parts of en.wikipedia.org. In contrast, discussion forums of online communities, where patients and physicians engage in discussions (often anonymously), have a colloquial language style, sometimes even slang. We tap into all three genres of text to demonstrate not only the applicability of our system, but also the amount of information buried in all of them. We use the Stanford CoreNLP software to preprocess all texts, such that they are tokenized, split into sentences, tagged with parts-of-speech, lemmatized, and parsed into syntactic dependency graphs.

**Table 2:** **Overview of KnowLife’s input corpus**

**Genre**	**Source**	**Documents**	**Sentences**
Scientific Publications	PubMed Medline	580,892	5,875,006
	PubMed Central	12,532	2,765,580
Encyclopedic Articles	Drugs.com	31,837	7,586,236
	Mayo Clinic	2,166	570,325
	Medline Plus	3,076	197,055
	RxList	2,515	1,102,791
	Wikipedia Health	20,893	787,148
Social Sources	Healthboards.com	752,778	37,270,371
	Patient.co.uk	44,610	1,081,420
	**Total**	**1,451,299**	**57,235,932**

### Entity recognition

The first stage in the KnowLife pipeline identifies sentences that may express a relational fact. We apply entity recognition to every sentence: a sentence with one or more entities is relevant for further processing. To efficiently handle the large dictionary and process large input corpora, we employ our own method [49], using string-similarity matching against the names in the UMLS dictionary. This method is two orders of magnitude faster than MetaMap [42], the most popular biomedical entity recognition tool, while maintaining comparable accuracy. Specifically, we use locality sensitive hashing (LSH) [50] with min-wise independent permutations (MinHash) [51] to quickly find matching candidates. LSH probabilistically reduces the high-dimensional space of all character-level 3-grams, while MinHash quickly estimates the similarity between two sets of 3-grams. A successful match provides us also with the entity’s semantic type. If multiple entities are matched to the same string in the input text, we currently do not apply explicit NED to determine the correct entity. Instead, using the semantic type hierarchy of UMLS, we select the most specifically typed entities. Later in the consistency reasoning stage, we leverage the type signatures to futher prune out mismatching entities. At the end of this processing stage, we have marked-up sentences such as

- *Anemia* is a common symptom of *sarcoidosis*.
- Eventually, a *heart attack* leads to *arrythmias*.
- Ironically, a *myocardial infarction* can also lead to *pericarditis*.

where *myocardial infarction* and *heart attack* are synonyms representing the same canonical entity.

### Pattern gathering

**Figure 2:**
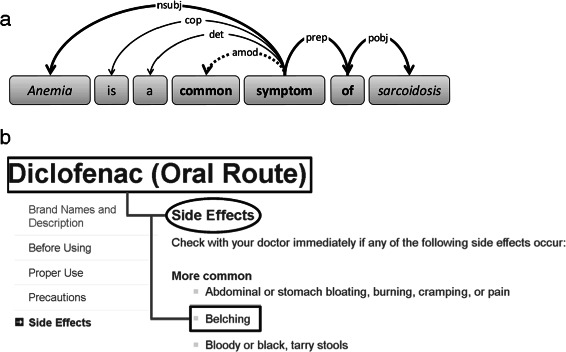
Pattern gathering in KnowLife.**(a)** Sentence-level pattern: Dependency graph of a sentence with recognized entities *anemia* and *sarcoidosis*. By computing the shortest path (bold lines) between the two entities, the word sequence *symptom of* is extracted. This sequence is extended by an adjectival modifier (amod) which results in the extracted pattern *common symptom of*. **(b)** Document-structure pattern: The entity *Diclofenac* is found within the document title and *Belching* within an <li> element. Take *Diclofenac* as the left-hand entity. By traversing the DOM tree downwards and coming across the heading *Side Effects*, we extract the heading’s text as a pattern. Further traversal leads us to *Belching*, which yields the right-hand entity for the pattern.

Our method extracts textual patterns that connect two recognized entities, either by the syntactic structure of a sentence or by a path in the DOM (Document Object Model) tree of a Web page. We extract two types of patterns: **Sentence-level Patterns:** For each pair of entities in a sentence, we extract a sequence of text tokens connecting the entities in the syntactic structure of the sentence. Specifically, this is the shortest path between the entities in the dependency graph obtained from parsing the sentence. However, this path does not necessarily contain the full information to deduce a relation; for instance, negations are not captured or essential adjectives are left out. Therefore, for every captured word the following grammatical dependencies are added: negation, adjectival modifiers, and adverbial modifiers. The resulting word sequence constitutes a sentence-level pattern. An example is shown in Figure 2(a). **Document-structure Patterns:** In Web portals like Mayo Clinic or Wikipedia, it is common that authors state medical facts by using specific document structures, like titles, sections, and listings. Such structures are encoded in the DOM tree of the underlying HTML markup. First, we detect if the document title, that is, the text within the <h1> tag in terms of HTML markup, is a single entity. Next, we detect if an entity appears in an HTML listing, that is, within an <li> tag. Starting from the <h1> tag, our method traverses the DOM tree downwards and determines all intermediate headings, i.e. <h2> to <h6> tags, until we reach the aforementioned <li> tag. The document title serves as left-hand entity, the intermediate headings as patterns, and the <li> text as right-hand entity. These are candidates for a relation or an entity argument in a relational fact. Figure 2(b) shows an example.

### Pattern analysis

The goal of the pattern analysis is to identify the most useful *seed patterns* out of all the pattern candidates gathered thus far. A seed pattern should generalize the over-specific phrases encountered in the input texts, by containing only the crucial words that express a relation and masking out (by a wildcard or part-of-speech tag) inessential words. This way we arrive at high-confidence patterns.

We harness the techniques developed in the Prospera tool [38]. First, an itemset mining algorithm is applied to find frequent sub-sequences in the patterns. The sub-sequences are weighed by statistical analysis, in terms of confidence and support. We use the seed facts and their co-occurrences with certain patterns as a basis to compute confidence, such that the confidence for a pattern *q* in a set of sentences *S* is defined as

$$\begin{array}{*{20}l} {}&confidence(q) =\\ {}&\frac{|\{s \in S ~|~ \exists (e_{1},e_{2}) \in SX(R_{i})\: \ q,e_{1},e_{2} \ occur \ in \ s\}|}{|\{s \in S ~|~ \exists (e_{1},e_{2}) \in SX(R_{i}) \cup CX(R_{i}) \: \ q,e_{1},e_{2} \ occur \ in \ s\}|} \end{array} $$

where *S* *X*(*R*_*i*_) is the set of all entity tuples (*e*_1_,*e*_2_) appearing in any seed fact with relation *R*_*i*_ and *C* *X*(*R*_*i*_) is the set of all entity tuples (*e*_1_,*e*_2_) appearing in any seed fact without relation *R*_*i*_. The rationale is that the more strongly a pattern correlates with the seed-fact entities of a particular relation, the more confident we are that the pattern expresses the relation. The patterns with confidence greater than a threshold (set to 0.3 in our experiments) are selected as seed patterns.

Each non-seed pattern *p* is then matched against the seed pattern set *Q* using Jaccard similarity to compute a weight *w* associating *p* with a relation.

$$w = max\{Jaccard(p, q) \times confidence(q) ~|~ q \in Q \} $$

The pattern occurrences together with their weights and relations serve as *fact candidates*. Table 3 shows sample seed patterns computed from seed facts. The table also gives examples for automatically acquired patterns and facts.

**Table 3:** **Examples of seed facts and seed patterns as well as automatically acquired patterns and facts**

**Seed facts**	**Seed patterns**	**Relations**	**Confidences**	**Patterns**	**Harvested facts**
*c* *a* *u* *s* *e* *s*(*T* *u* *b* *e* *r* *c* *u* *l* *o* *s* *i* *s*,*P* *e* *r* *i* *c* *a* *r* *d* *i* *t* *i* *s*)	progress	createsRisk	0.5	which progresses to	*c* *a* *u* *s* *e* *s*(*P* *e* *r* *i* *c* *a* *r* *d* *i* *t* *i* *s*,*T* *a* *m* *p* *o* *n* *a* *d* *e*)
*c* *r* *e* *a* *t* *e* *s* *R* *i* *s* *k*(*O* *b* *e* *s* *i* *t* *y*,*D* *i* *a* *b* *e* *t* *e* *s*)		causes	0.5	still progressing to	*c* *r* *e* *a* *t* *e* *s* *R* *i* *s* *k*(*W* *a* *r* *t*,*S* *k* *i* *n* *c* *a* *r* *c* *i* *n* *o* *m* *a*)
*c* *r* *e* *a* *t* *e* *s* *R* *i* *s* *k*(*O* *b* *e* *s* *i* *t* *y*,*A* *s* *t* *h* *m* *a*)	risk factor	createsRisk	1.0	children risk factors	*c* *r* *e* *a* *t* *e* *s* *R* *i* *s* *k*(*W* *o* *o* *d* *D* *u* *s* *t*,*A* *s* *t* *h* *m* *a*)
*c* *r* *e* *a* *t* *e* *s* *R* *i* *s* *k*(*M* *a* *l* *a* *r* *i* *a*,*S* *t* *i* *l* *l* *b* *i* *r* *t* *h*)				have risk factors	*c* *r* *e* *a* *t* *e* *s* *R* *i* *s* *k*(*G* *o* *l* *f*,*T* *e* *n* *d* *i* *n* *i* *t* *i* *s*)
				known risk factors	*c* *r* *e* *a* *t* *e* *s* *R* *i* *s* *k*(*G* *B* *v* *i* *r* *u* *s* *C*,*H* *e* *p* *a* *t* *i* *t* *i* *s*)
*i* *s* *S* *y* *m* *p* *t* *o* *m*(*P* *a* *i* *n*,*C* *r* *o* *h* *n* *D* *i* *s* *e* *a* *s* *e*)	occur	affects	0.67	occurs anywhere	*a* *f* *f* *e* *c* *t* *s*(*H* *a* *s* *h* *i* *m* *o* *t* *o* ^′^ *s*,*T* *h* *y* *r* *o* *i* *d* *G* *l* *a* *n* *d*)
*a* *f* *f* *e* *c* *t* *s*(*P* *e* *r* *i* *c* *a* *r* *d* *i* *t* *i* *s*,*H* *e* *a* *r* *t*)		isSymptom	0.33	occurs patients	*i* *s* *S* *y* *m* *p* *t* *o* *m*(*A* *n* *e* *m* *i* *a*,*S* *a* *r* *c* *o* *i* *d* *o* *s* *i* *s*)

### Consistency reasoning

The pattern analysis stage provides us with a large set of fact candidates and their supporting patterns. However, these contain many false positives. To prune these out and improve precision, the last stage of KnowLife applies logical consistency constraints to the fact candidates and accepts only a consistent subset of them.

We leverage two kinds of manually defined semantic constraints: i) the type signatures of relations (see Table 1) for type checking of fact candidates, and ii) mutual exclusion constraints between certain pairs of relations. For example, if a drug has a certain symptom as a side effect, it cannot treat this symptom at the same time. These rules allow us to handle conflicting candidate facts. The reasoning uses probabilistic weights derived from the statistics of the candidate gathering phase.

To reason with consistency constraints, we follow the framework of [37], by encoding all facts, patterns, and grounded (i.e., instantiated) constraints into weighted logical clauses. We extend this prior work by computing informative weights from the confidence statistics obtained in the pattern-based stage of our IE pipeline. We then use a weighted Max-Sat solver to reason on the hypotheses space of fact candidates, to compute a consistent subset of clauses with the largest total weight. Due to the NP-hardness of the weighted Max-Sat problem, we resort to an approximation algorithm that combines the dominating-unit-clause technique [52] with Johnson’s heuristic algorithm [53]. Suchanek et al. [37] has shown that this combination empirically gives very good approximation ratios. The complete set of consistency constraints is in the supplementary material (see Additional file 1).

## Results and discussion

We ran extensive experiments with the input corpora listed in Table 2, and created different KB’s based on different configurations. We assess the size and quality of each KB, in terms of their numbers of facts and their precision evaluated by random sampling of facts. Tables 4 and 5 give the results, for different choices of input corpora and different configurations of the KnowLife pipeline, respectively. Recall is not evaluated, as there is no gold standard for fully comprehensive facts. To ensure that our findings are significant, for each relation, we computed the Wilson confidence interval at *α* = 5%, and kept evaluating facts until the interval width fell below 5%. An interval width of 0% means that all the facts were evaluated. Four different annotators evaluated the facts, judging them as true or false based on provenance information. As for inter-annotator agreement, 22,002 facts were evaluated; the value of Fleiss’ Kappa was 0.505, which indicates a moderate agreement among all four annotators. The complete set of evaluated facts is in the supplementary material (see Additional file 3).

**Table 4:** **Evaluation of different text genres**

**Relation**	**Precision**				**Harvested facts**			
	**Encyclopedic**	**Scientific**	**Encyclopedic +**	**Encyclopedic +**	**Encyclopedic**	**Scientific**	**Encyclopedic +**	**Encyclopedic +**
	**sources**	**sources**	**scientific**	**scientific +**	**sources**	**sources**	**scientific**	**scientific +**
			**sources**	**social sources**			**sources**	**social sources**
Affects	0.855 ±0.047	0.762 ±0.049	**0.825 ±0.047**	0.767 ±0.048	1,278	450	**2,388**	5,053
Aggravates	0.810 ±0.041	0.459 ±0.044	**0.829 ±0.049**	0.785 ±0.049	130	371	**432**	708
Alleviates	0.953 ±0.039	0.735 ±0.048	**0.786 ±0.046**	0.736 ±0.048	903	4,433	**4,530**	6,790
Causes	0.904 ±0.039	0.674 ±0.049	**0.801 ±0.049**	0.792 ±0.049	28,119	19,203	**47,463**	62,407
Complication	0.917 ±0.039	0.397 ±0.049	**0.897 ±0.041**	0.869 ±0.046	1,011	1,475	**1,524**	1,566
Contraindicates	0.874 ±0.048	0.710 ±0.000	**0.961 ±0.030**	0.908 ±0.048	512	49	**1,808**	1,831
CreatesRisk	0.878 ±0.047	0.569 ±0.049	**0.720 ±0.040**	0.620 ±0.049	4,407	24,695	**18,508**	32,211
Diagnoses	0.964 ±0.035	0.839 ±0.049	**0.860 ±0.048**	0.840 ±0.047	813	5,920	**4,832**	9,743
Interacts	0.964 ±0.035	0.709 ±0.000	**0.965 ±0.034**	0.957 ±0.034	164,912	103	**164,912**	164,912
IsSymptom	0.891 ±0.042	0.482 ±0.050	**0.858 ±0.048**	0.694 ±0.048	4,878	2,320	**6,395**	11,017
ReducesRisk	0.797 ±0.045	0.637 ±0.046	**0.762 ±0.048**	0.751 ±0.049	1,712	4,684	**4,489**	5,865
SideEffect	0.956 ±0.038	0.826 ±0.000	**0.964 ±0.035**	0.971 ±0.026	270,600	139	**270,709**	271,416
Treats	0.850 ±0.048	0.581 ±0.045	**0.898 ±0.041**	0.566 ±0.048	11,915	9,318	**14,699**	35,803
Aggregated ^∗^	0.951	0.630	**0.933**	0.892	491,190	73,160	**542,689**	609,322

*Precision values are averaged and numbers of harvested facts are summed.

**Table 5:** **Evaluation of the impact of different components**

**Relation**	**Precision**				**Harvested facts**			
	**Full pipeline**	**Without**	**Without**	**Without**	**Full pipeline**	**Without**	**Without**	**Without**
	**encyclopedic +**	**document**	**statistical**	**consistency**	**encyclopedic +**	**document**	**statistical**	**consistency**
	**scientific sources**	**structure**	**analysis**	**reasoning**	**scientific sources**	**structure**	**analysis**	**reasoning**
Affects	0.825 ±0.047	0.882 ±0.044	0.821 ±0.048	0.171 ±0.051	2,388	2,350	4,088	29,477
Aggravates	0.829 ±0.049	0.833 ±0.036	0.598 ±0.049	0.592 ±0.053	432	431	592	1,730
Alleviates	0.786 ±0.046	0.778 ±0.050	0.320 ±0.049	0.289 ±0.062	4,530	4,387	18,142	16,943
Causes	0.801 ±0.049	0.800 ±0.046	0.631 ±0.048	0.490 ±0.069	47,463	30,563	66,833	91,784
Complication	0.897 ±0.041	0.781 ±0.048	0.376 ±0.050	0.739 ±0.050	1,524	700	4,812	2,955
Contraindicates	0.961 ±0.030	0.914 ±0.043	0.122 ±0.049	0.630 ±0.059	1,808	365	26,298	15,279
CreatesRisk	0.720 ±0.040	0.750 ±0.044	0.386 ±0.047	0.406 ±0.067	18,508	17,282	77,158	48,159
Diagnoses	0.860 ±0.048	0.887 ±0.044	0.802 ±0.049	0.303 ±0.063	4,832	4,002	7,467	35,326
Interacts	0.965 ±0.034	0.858 ±0.046	0.953 ±0.047	0.941 ±0.049	164,912	392	200,935	187,201
IsSymptom	0.858 ±0.048	0.691 ±0.050	0.625 ±0.049	0.328 ±0.064	6,395	2,920	9,543	29,776
ReducesRisk	0.762 ±0.048	0.729 ±0.050	0.228 ±0.046	0.406 ±0.067	4,489	4,043	11,023	14,729
SideEffect	0.964 ±0.035	0.938 ±0.048	0.941 ±0.046	0.879 ±0.050	270,709	924	270,427	338,645
Treats	0.898 ±0.041	0.784 ±0.050	0.549 ±0.050	0.402 ±0.067	14,699	14,057	23,473	45,439
Aggregated ^∗^	0.933	0.784	0.777	0.707	542,689	82,416	720,791	857,443

*Precision values are averaged and numbers of harvested facts are summed.

### Impact of different text genres

We first discuss the results obtained from the different text genres: i) scientific (PubMed publications), ii) encyclopedic (Web portals like Mayo Clinic or Wikipedia), iii) social (discussion forums). Table 4 gives, column-wise, the number of facts and precision figures for four different combinations of genres.

Generally, combining genres gave more facts at a lower precision, as texts of lower quality like social sources introduced noise. The combination that gave the best balance of precision and total yield was scientific with encyclopedic sources, with a micro-averaged precision of 0.933 for a total of 542,689 facts. We consider this the best of the KB’s that KnowLife generated.

The best overall precision was achieved when using encyclopedic texts only. This confirmed our hypothesis that a pattern-based approach works best when the language is simple and grammatically correct. Contrast this with scientific publications which often exhibit convoluted language, and online discussions with a notable fraction of grammatically incorrect language. In these cases, the quality of patterns degraded and precision dropped. Incorrect facts stemming from errors in the entity recognition step were especially rampant in online discussions, where colloquial language (for example, *meds*, or short for *medicines*) led to incorrect entities (acronym for *Microcephaly, Epilepsy, and Diabetes Syndrome*).

The results vary highly across the 13 relations in our experiments. The number of facts depends on the extent to which the text sources express a relation, while precision reflects how decisively patterns point to that relation. *Interacts* and *SideEffect* are prime examples: the drugs.com portal lists many side effects and drug-drug interactions by the DOM structure, which boosted the extraction accuracy of KnowLife, leading to many facts at precisions of 95.6% and 96.4%, respectively. Facts for the relations *Alleviates*, *CreatesRisk*, and *ReducesRisk*, on the other hand, mostly came from scientific publications, which resulted in fewer facts and lower precision.

A few relations, however, defied these general trends. Patterns of *Contraindicates* were too sparse and ambiguous within encyclopedic texts alone and also within scientific publications alone. However, when the two genres were combined, the good patterns reached a critical mass to break through the confidence threshold, giving rise to a sudden increase in harvested facts. For the *CreatesRisk* and *ReducesRisk* relations, combining encyclopedic and scientific sources increased the number of facts compared to using only encyclopedic texts, and increased the precision compared to using only scientific publications.

As Table 4 shows, incorporating social sources brought a significant gain in the number of harvested facts, at a trade-off of lowered precision. As [46] pointed out, there are facts that come only from social sources and, depending on the use case, it is still worthwhile to incorporate them; for example, to facilitate search and discovery applications where recall may be more important. Morever, the patterns extracted from encyclopedic and scientific sources could be reused to annotate text in social sources, so as to identify existing information.

Taking a closer look at the best experimental setting, we see that scientific and encyclopedic sources in KnowLife contribute to a different extent to the number of harvested facts. Table 6 shows the number of fact occurrences in our input sources. Recall that a fact can occur in multiple sentences in multiple text sources. Our experiments show that encyclopedic articles are more amenable for harvesting facts than scientific publications.

**Table 6:** **Number of fact occurrences in text sources**

**Genre**	**Source**	**Fact occurrences**
Scientific Publications	PubMed Medline	39,266
	PubMed Central	6,979
Encyclopedic Articles	Drugs.com	461,130
	Mayo Clinic	35,300
	Medline Plus	6,559
	RxList	5,818
	Wikipedia Health	17,588

### Impact of different components

In each setting, only one component was disabled, and the processing pipeline ran with all other components enabled. We used the KnowLife setting with scientific and encyclopedic sources, which, by and large, performed best, as the basis for investigating the impact of different components in the KnowLife pipeline. To this end, we disabled individual components: DOM tree patterns, statistical analysis of patterns, consistency reasoning – each disabled separately while retaining the others. This way we obtained insight into how strongly KnowLife depends on each component. Table 5 shows the results of this ablation study.

**No DOM tree patterns:** When disregarding patterns on the document structure and solely focusing on textual patterns, KnowLife degrades in precision (from 93% to 78%) and sharply drops in the number of acquired facts (from ca. 540,000 to 80,000). The extent of these general effects varies across the different relations. Relations whose patterns are predominantly encoded in document structures – once again *Interacts* and *SideEffect* – exhibit the most drastic loss. On the other hand, relations like *Affects*, *Aggravates*, *Alleviates*, and *Treats*, are affected only to a minor extent, as their patterns are mostly found in free text.

**No statistical pattern analysis:** Here we disabled the statistical analysis of pattern confidence and the frequent itemset mining for generalizing patterns. This way, without confidence values, KnowLife kept all patterns, including many noisy ones. Patterns that would be pruned in the full configuration led to poor seed patterns; for example, the single word *causes* was taken as a seed pattern for both relations *SymptomOf* and *Contraindicates*. Without frequent itemset mining, long and overly specific patterns also contributed to poor seed patterns. The combined effect greatly increased the number of false positives, thus dropping in precision (from 93% to 77%). In terms of acquired facts, not scrutinizing the patterns increased the yield (from ca. 540,000 to 720,000 facts).

Relations mainly extracted from DOM tree patterns, such as *Interacts* and *SideEffect*, were not much affected. Also, relations like *Affects* and *Diagnoses* exhibited only small losses in precision; for these relations, the co-occurrence of two types of entities is often already sufficient to express a relation. The presence of consistency constraints on type signatures also helped to keep the output quality high.

**No consistency reasoning:** In this setting, neither type signatures nor other consistency constraints were checked. Thus, conflicting facts could be accepted, leading to a large fraction of false positives. This effect was unequivocally witnessed by an increase in the number of facts (from ca. 540,000 to 850,000) accompanied by a sharp decrease in precision (from 93% to 70%).

The relations *Interacts* and *SideEffect* were least affected by this degradation, as they are mostly expressed in the via document structure of encyclopedic texts where entity types are implicitly encoded in the DOM tree tags (see Figure 2). Here, consistency reasoning was not vital.

**Lessons learned:** Overall, this ablation study clearly shows that all major components of the KnowLife pipeline are essential for high quality (precision) and high yield (number of facts) of the constructed KB. Each of the three configurations where one component is disabled suffered substantial if not dramatic losses in either precision or acquired facts, and sometimes both. We conclude that the full pipeline is a well-designed architecture whose strong performance cannot be easily achieved by a simpler approach.

### Error analysis

We analyzed the causes of error for all 760 facts annotated as incorrect from the experimental setting using the full information extraction pipeline and all three text genres. This setting allows us to compare the utility of the different components as well as the different genres. As seen in Table 7, we categorize the errors as follows: **Preprocessing:** At the start of the pipeline, incorrect sentence segmentation divided a text passage into incomplete sentences, or left multiple sentences undivided. This in turn lead to incorrect parsing of syntactic dependency graphs. In addition, there were incorrectly parsed DOM trees in Web portal documents. Not surprisingly, almost all preprocessing errors came from encyclopedic and social sources due to their DOM tree structure and poor language style, respectively. **Entity Recognition:** Certain entities were not correctly recognized. Complex entities are composed of multiple simple entities; examples include *muscle protein breakdown* recognized as *muscle protein* and *breakdown*, or *arrest of cystic growth* recognized as *arrest* and *cystic growth*. Paraphrasing and misspelling entities cause their textual expressions to deviate from dictionary entries. Idiomatic expressions were incorrectly picked up as entities. For instance, there is no actual physical activity in the English idiom *in the long run*. **Entity Disambiguation:** Selecting an incorrect entity out of multiple matching candidates caused this error, primarily due to two reasons. First, the type signatures of our relations were not sufficient to futher prune out mismatching entities during fact extraction. Second, colloquial terms not curated in the UMLS dictionary were incorrectly resolved. For example, *meds* for medicines was disambiguated as the entity *Microcephaly, Epilepsy, and Diabetes Syndrome*. **Coreferencing:** Due to the lack of coreference resolution, correct entities were obscured by phrases such as *this protein* or *the tunnel structure*. **Nonexistent relation:** Two entities might co-occur within the same sentence without sharing a relation. When a pattern occurrence between such entities was nevertheless extracted, it resulted in an unsubstantiated relation. **Pattern Relation Duality:** A pattern that can express two relations was harvested but assigned to an incorrect relation. For example, the pattern *mimic* was incorrectly assigned to the relation *isSymptom*. **Swapped left and right-hand entity:** The harvested fact was incorrect because the left- and right-hand entities were swapped. Consider the example fact *i* *s* *S* *y* *m* *p* *t* *o* *m*(*A* *n* *e* *m* *i* *a*,*S* *a* *r* *c* *o* *i* *d* *o* *s* *i* *s*), which can be expressed by either sentence:

1. Anemia is a common symptom of sarcoidosis.

**Table 7:** **Error analysis (number of facts in brackets)**

		**Percentage based on text genre**		
**Percentage**	**Cause of error**	**Encyclopaedic**	**Scientific**	**Social**
		**sources**	**sources**	**sources**
8.16% (62)	Preprocessing	38.71% (24)	3.23% (2)	58.06% (36)
27.24% (207)	Entity Recognition	13.04% (27)	45.41% (94)	41.55% (86)
32.11% (244)	Entity Disambiguation	12.30% (30)	26.23% (64)	61.48% (150)
1.97% (15)	Coreferencing	13.33% (2)	13.33% (2)	73.33% (11)
13.68% (104)	Nonexistent Relation	23.08% (24)	29.81% (31)	47.12% (49)
9.21% (70)	Pattern Relation Duality	24.29% (17)	27.14% (19)	48.57% (34)
3.29% (25)	Swapped left and right-hand entity	28.00% (7)	24.00% (6)	48.00% (12)
3.03% (23)	Negation	17.39% (4)	21.74% (5)	60.87% (14)
1.32% (10)	Factually Wrong	40.00% (4)	10.00% (1)	50.00% (5)
2. A common symptom of sarcoidosis is anemia.

In both cases, the same pattern *is a common symptom of* is extracted. In sentence 2, however, an incorrect fact would be extracted since the order in which the entities occur is reversed. **Negation:** This error was caused by not detecting negation expressed in the text. The word expressing the negation may occur textually far away from the entities, as in *It is disputed whether early antibiotic treatment prevents reactive arthritis*, and thus escaped our pattern gathering method. In other cases, the negation phrase will require subtle semantic understanding to tease out, as in *Except for osteoarthritis, I think my symptoms are all from heart disease*. **Factually Wrong:** Although our methods successfully harvested a fact, the underlying text evidence made a wrong statement. **Lessons learned:** Overall, this error analysis confirms that scientific and encyclopedic sources contain well-written texts that are amenable to a text mining pipeline. Social sources, with their poorer quality of language style as well as information content, were the biggest contributor in almost all error categories. Errors in entity recognition and disambiguation accounted for close to 60% of all errors; overcoming them will require better methods that go beyond a dictionary, and incorporate deeper linguistic and semantic understanding.

### Coverage

The overriding goal of KnowLife has been to create a versatile KB that spans many areas within the life sciences. To illustrate which areas are covered by KnowLife, we refer to the *semantic groups* defined by [54]. Table 8 shows the number of acquired facts for pairs of the thirteen different areas inter-connected in our KB. This can be seen as an indicator that we achieved our goal at least to some extent.

**Table 8:** **Top-20 pairs of inter-connected biomedical areas within KnowLife**

**Biomedical areas**		**Connections**
Disorders	Chemicals	310482
Chemicals	Chemicals	190160
Disorders	Disorders	36677
Disorders	Procedures	14169
Chemicals	Physiology	5397
Disorders	Genes	3831
Disorders	Living Beings	2539
Chemicals	Drugs	2455
Disorders	Anatomy	2895
Disorders	Devices	792
Disorders	Activities	592
Disorders	Drugs	511
Disorders	Objects	505
Chemicals	Procedures	544
Disorders	Physiology	370
Procedures	Physiology	123
Procedures	Living Beings	99
Disorders	Geographical Areas	82
Genes	Physiology	51
Disorders	Phenomena	50

The predominant number of facts involves entities of the semantic group *Disorders*, for two reasons. First, with our choice of relations, disorders appear in almost all type signatures. Second, entities of type clinical finding are covered by the group *Disorders*, and these are frequent in all text genres. However, this type also includes diverse, non-disorder entities such as *pregnancy*, which is clearly not a disorder.

## Conclusions

### Application benefit

To showcase the usefulness of KnowLife, we developed a health portal (http://knowlife.mpi-inf.mpg.de) that allows interactive exploration of the harvested facts and their input sources. The KnowLife portal supports a number of use cases for different information needs [48]. A patient may wish to find out the side effects of a specific drug, by searching for the drug name and browsing the *SideEffect* facts and their provenance. A physician may want to “speed read” publications and online discussions on treatment options for an unfamiliar disease. Provenance information is vital here, as the physician would want to consider the recency and authority of the sources for certain statements. The health portal also provides a function for on-the-fly annotation of new text from publications or social media, leveraging known patterns to highlight any relations found.

### Future work

In the future, we plan to improve the entity recognition to accommodate a wider variety of entities beyond those in UMLS. For instance, colloquial usage (*meds* for *medicines*) and composite entities (*amputation of right leg*) are not yet addressed. Entities within UMLS also require more sophisticated disambiguation. For instance, the text occurrence *stress* may be correctly distinguished between the brand name of a drug and the psychological feeling.

Finally, we would like to address the challenge of mining and representing the context of harvested facts. Binary relations are often not sufficient to express medical knowledge. For example, the statement *Fever is a symptom of Lupus Flare during pregnancy* cannot be suitably represented by a binary fact.

We plan to cope with such statements by extracting ternary and higher-arity relations, with appropriate extensions of both pattern-based extraction and consistency reasoning.

## Additional files

Additional file 1
**ConsistencyRules.** A collection of consistency rules employed during the reasoning stage.

Additional file 2
**TypeSignature.** A list of our relations and their type signatures, which are UMLS semantic type names.

Additional file 3
**EvaluationResults.** A table covering all evaluated facts, i.e. left-hand argument, relation, right-hand argument, and human judgment (majority vote).
